# Increased interleukin-27 cytokine expression in the central nervous system of multiple sclerosis patients

**DOI:** 10.1186/s12974-017-0919-1

**Published:** 2017-07-24

**Authors:** Patrice H. Lalive, Mario Kreutzfeldt, Odile Devergne, Imke Metz, Wolfgang Bruck, Doron Merkler, Caroline Pot

**Affiliations:** 1 Unit of Neuroimmunology and Neuromuscular Diseases, Division of Neurology, Department of Clinical Neurosciences, Geneva University Hospital, 1211 Geneva 4, Switzerland; 2 Department of Pathology and Immunology, Geneva University Hospital and University of Geneva, 1211 Geneva 4, Switzerland; 3 Department of Pathology and Immunology, Clinical Pathology Division, Geneva University Hospital and University of Geneva, 1211 Geneva 4, Switzerland; 4 Institut Necker Enfants Malades, INSERM U1151 CNRS UMR 8253, Université Paris Descartes, Sorbonne Paris Cité, 75 015 Paris, France; 5 Institute of Neuropathology, University Medical Center Göttingen, 37075 Göttingen, Germany; 6 Laboratories of Neuroimmunology, Division of Neurology and Neuroscience Research Center, Department of Clinical Neurosciences, Lausanne University Hospital, 1011 Lausanne, Switzerland

**Keywords:** Cytokines, Interleukin-27, Multiple sclerosis, Cerebrospinal fluid, Immunohistochemistry

## Abstract

**Background:**

Multiple sclerosis (MS) is an autoimmune disorder characterized by chronic inflammation, demyelination, and neuronal damage. During autoimmunity, cytokines are important mediators of the inflammation. In this line, interleukin-27 (IL-27) modulates inflammation and can be produced directly at inflammatory sites such as in the joints during rheumatoid arthritis or in the central nervous system (CNS) during MS. While in animal models of MS, treatment with IL-27 decreases the disease severity, its role in humans is not clearly established and it is not known if IL-27 could be detected in the cerebrospinal fluid (CSF) of MS patients.

**Methods:**

In this study, we measured IL-27 levels using a quantitative enzyme-linked immunosorbent assay in CSF of patients with relapsing remitting multiple sclerosis (RRMS), isolated optic neuritis (ON) and non-inflammatory neurological disease (NIND) as well as in the sera of healthy donors (HD) and RRMS patients undergoing different disease modifying treatments. We further confirmed by immunohistology of patient biopsies the identity of IL-27 producing cells in the brain of active MS lesions.

**Results:**

We observed that IL-27 levels are increased in the CSF but not in the sera of RRMS compared to HD. We confirmed that IL-27 is expressed in active MS plaques by astrocytes of MS patients.

**Conclusions:**

Our results point toward a local secretion of IL-27 in the CNS that is increased during autoimmune processes. We propose that local production of IL-27 could sign the induction of a regulatory response that promotes inflammation’s resolution. The effect of new immunomodulatory therapies on cerebral IL-27 production could be used to understand the biology of IL-27 in MS disease.

## Background

Multiple sclerosis (MS) is an autoimmune disorder characterized by demyelination, chronic inflammation, and neuronal damage. Among mediators recognized to contribute to inflammatory processes in the CNS, accumulating evidence indicates that members of interleukin-12 (IL-12) family of cytokines, which includes IL-12, IL-23, IL-35, and IL-27, are important mediators of inflammation. While IL-12 and IL-23 have clearly been demonstrated to be pro-inflammatory, IL-27 has been ascribed with additional anti-inflammatory properties. In this line, continuous infusion of IL-27 has been shown to dampen disease severity during the experimental autoimmune encephalomyelitis (EAE), an animal model for MS [1–3]. In addition, mice that lack the receptor for IL-27 or IL-27-downstream signaling transcription factors are characterized by more severe EAE disease compared to control mice [4, 5]. Translational human studies performed in vitro indicate that IL-27 secretion from dendritic cells (DCs) obtained from healthy donors can increase upon exposure to the drug-modifying treatment IFN-β. Further, in vitro analysis proposed IL-27 as a marker to identify responders to IFN-β treatment [6] as IL-27 secretion was increased from ex vivo isolated plasmacytoid dendritic cells (*pDCs*) from MS patients treated with IFN-β for 1 month [7]. In this line, elevation of IL-27 has been observed in patients responding to glatiramer acetate therapy [8]. Those studies all point toward an anti-inflammatory function of IL-27 during CNS autoimmunity.

In addition to IL-27 secretion in the immune peripheral compartment, IL-27 can be produced directly at the sites of inflammation by local resident cells and antigen presenting cells (APC). In rheumatoid arthritis, increased IL-27 levels in the joint synovial fluid but not in the blood have been reported [9]. Furthermore, IL-27 can be secreted by resident cells of the CNS such as astrocytes or microglia cultured in vitro [10, 11]. IL-27 was shown to be expressed in astrocytes and microglia in MS brains [12]. However, it is not known if IL-27 could be detected directly in the CSF of RRMS patients. We here show that IL-27 production from the CNS can be directly assessed in CSF and in addition that it is increased in RRMS compared to other non-inflammatory neurological diseases or healthy controls. We further confirm that IL-27 is expressed in astrocytes from brain biopsies of MS patients presenting with active lesions.

## Methods

### CSF and sera

Cerebrospinal fluids (CSF), collected in excess during spinal taps performed for diagnosis purposes, were stored anonymously in a centralized biobank for retrospective research purposes. This research was conducted according to the regulations of the Act on Medical Research Involving Human Subjects of the Geneva University Hospital (HUGO.RE.DG.0010). This study was approved by The Geneva University Hospital Medical Ethical board (Authorization # 07-261R (NAC 07-102R).

The three CSF groups tested were composed of relapsing remitting multiple sclerosis (RRMS), isolated optic neuritis (ON), and non-inflammatory neurological diseases (NIND). NIND consisted of patients who underwent spinal tap for investigation of peripheral seventh nerve palsy or of headaches, which revealed to be non-inflammatory. For RRMS patients, the revised 2005 McDonald Criteria [13] were used to diagnose MS. Demographic and clinical characteristics, including gender, age, neurological symptoms and signs, and CSF white blood cell (WBC) count were retrieved from our CSF biobank database as previously described [14].

Sera were collected from healthy controls (HC) and RRMS patients under different disease-modifying treatments.

### Analysis of IL-27 in the sera and CSF

IL-27 was measured using an enzyme immune-assay (Human IL-27 ELISA, Kit: Duo Set (R&D) Ref:DY2526).

### EBI3 immunohistochemistry

Post-mortem samples and biopsies from the human MS brain were assessed. Formalin-fixed paraffin-embedded tissue was cut in 2-μm-thick sections. Sections were dewaxed, rehydrated, and subjected to antigen retrieval by heat pre-treatment using citrate buffer pH 6. Ebi3 antibody (clone 2G4H6, mouse monoclonal antibody IgG2a) was diluted 1:500 if visualized using EnVision^+^ kit (DAKO) or 1:200 together with AffiniPure Goat Anti-Mouse IgG (H+L) AlexaFluor488 labeled antibody (Jackson Immunoresearch) for immunofluorescence. GFAP (DAKO, Z0334, rabbit) antibodies were directly labeled with AlexaFluor555 or AlexaFluor647 following the protocol of the antibody labeling kit (Invitrogen) and applied 1:100. Nuclei were counterstained by H&E for brightfield or by DAPI for fluorescence. Slides were acquired using the Panoramic 250 Flash II (3D-Histech) slide scanner.

### Statistical analysis

Differences in variables were analyzed using ANOVA and Student’s *t* tests (for normally distributed data) or Kruskal–Wallis and Mann–Whitney *U* tests (for non-normally distributed data) as appropriate.

## Results

### Increased production of IL-27 in CSF during RRMS is correlated with disease activity

**Fig. 1:**
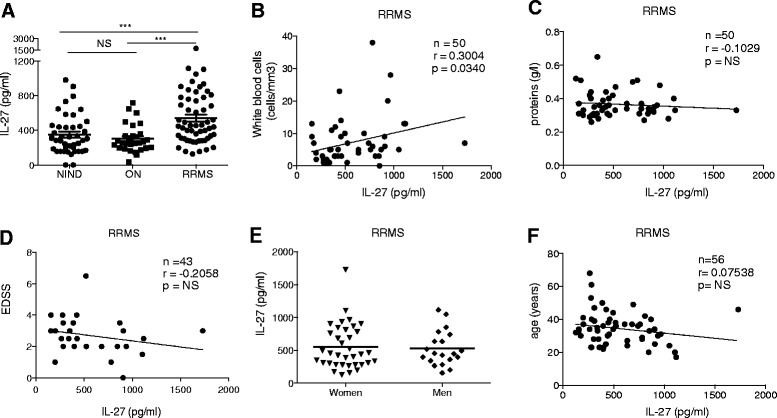
Distribution of CSF IL-27 levels in patient subgroups. **a** The CSF IL-27 levels of all individual patients were measured using quantitative ELISA. Data are expressed in pg/mL. Only significant *p* values are shown in the graph (*p* < 0.001; Kruskal–Wallis test). *ON* optic neuritis, *RRMS* relapsing remitting multiple sclerosis, *NIND* non-inflammatory neurological diseases. Correlation between IL-27 and **b** white blood cells counts, **c** total proteins levels in the CSF, **d** EDSS score, **e** gender, and **f** age. Pearson product-moment correlation coefficients (*r*) and *p* values are shown

Astrocytes have recently been proposed as a source of IL-27 secretion in active MS lesions [12], which would suggest that IL-27 is directly produced at the site of inflammation in the central nervous system. We therefore examined IL-27 secretion in CSF. Using the ELISA that specifically detects the cytokine IL-27, CSF concentrations of IL-27 were measured in untreated patients with RRMS (*n* = 56), with ON (*n* = 29), or with NIND (*n* = 42) (Fig. 1). The demographic feature characteristics of patients are shown in Table 1. NIND consisted of patients who underwent spinal tap for investigation of peripheral seventh nerve palsy (*n* = 13) or of headaches (*n* = 29), which revealed to be non-infectious and non-inflammatory.

**Table 1:** Demographic and clinical characteristics of patients (CSF samples *n* = 127)

	NIND (*n* = 42)	ON (*n* = 29)	RRMS (*n* = 56)
Age (years), mean (±SD)	38.38 (±13.42)	33.03 (±11.72)	34.7 (±9.994)
Gender			
Women	20 (48%)	23 (80%)	35 (62.5%)
Men	22 (52%)	6 (20%)	21 (37.5%)
EDSS, mean	–	–	2.895 (±1.485)
Treatments			
DMT	–	0	0
Corticoids	–	0	2 (3.5%)

*NIND* non inflammatory neurological disease, *ON* optic neuritis, *RRMS* relapsing remitting multiple sclerosis, *EDSS* expanded disability status scale, *DMT* disease-modifying treatments

Our results indicate a higher production of IL-27 in the CSF of RRMS patients compared to isolated ON and NIND (*p* < 0.005) (Fig. 1a). The spinal tap was performed at the time of diagnosis; therefore, none of the patients were under a disease-modifying treatment and only two patients had received corticoid treatment. The levels of IL-27 for those two patients were in the range of the other values.

Significant correlation between IL-27 levels and WBC counts (Fig. 1b) but not with protein levels (Fig. 1c) were observed in the CSF of RRMS patients. Those results indicate a link between CNS inflammation and a local IL-27 secretion and argue against increased CSF IL-27 levels through blood-brain-barrier (BBB) leakage. The CSF levels of IL-27 were correlated to neither disease severity as indicated by the EDSS score (Fig. 1d) nor gender (Fig. 1e) nor age (Fig. 1f).

### IL-27 CSF levels are correlated to oligoclonal bands positivity

**Fig. 2:**
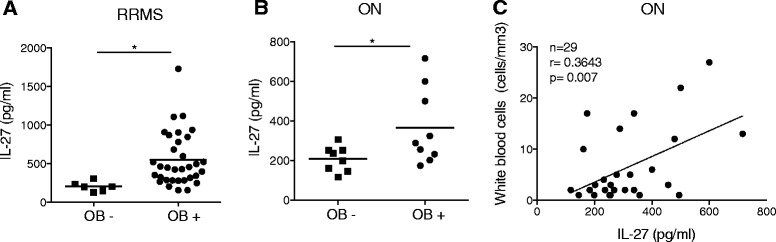
Distribution of CSF IL-27 levels is correlated with positivity in oligoclonal IgG bands (OB). CSF IL-27 levels and OB pattern in **a** RRMS and **b** ON individual patients. **c** Correlation between IL-27 and white blood cells counts in ON patients

The correlation between inflammatory parameters and IL-27 levels prompted us to further look at the correlation between oligoclonal IgG bands (OB) profile in the CSF and IL-27 levels. OB accompany CNS inflammation and are found in 95% of patients with MS. Interestingly, IL-27 CSF levels were lower in the few RRMS patients that did not present OB positivity (Fig. 2a). Those results lead us to explore the correlation between OB and IL-27 levels in ON patients. Although IL-27 levels were not elevated in ON patients compared to HC, ON patients with a positive OB pattern (that are at higher risk to develop MS) showed significant higher levels of IL-27 compared with ON patients with a negative OB pattern (Fig. 2b). Similar to RRMS patients, we noted in the CSF of ON patients a significant correlation between IL-27 levels and WBC (Fig. 2c), but not with protein levels (data not shown).

### No significant change in IL-27 expression in the sera of RRMS-treated patients

**Fig. 3:**
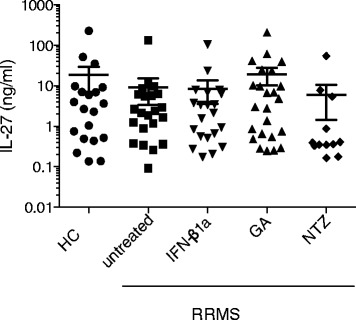
IL-27 levels are similar in sera of healthy controls and RRMS patients. Sera were obtained from aged-matched healthy controls (HC) and patients with RRMS without treatment (untreated) or treated with interferon beta1a (INF-β1a), glatiramer acetate (GA), or natalizumab (NTZ)

In order to assess if the protein levels of IL-27 were increased in the immune peripheral compartment, IL-27 levels were measured by ELISA in the sera of HC donors (*n* = 23) and RRMS patients without treatment (*n* = 30) or treated with Interferon beta (IFN-β1a, *n* = 28), glatiramer acetate (GA, *n* = 24), or natalizumab (NTZ, *n* = 12). The demographic features and clinical characteristics of patients are shown in Table 2. By contrast to the CSF, IL-27 sera levels of RRMS patients were equivalent to those observed in HC and were not significantly influenced by disease-modifying treatments (Fig. 3).

**Table 2:** Demographic characteristics of patients for serum analysis

	CTRL (*n* = 20)	Untreated (*n* = 22)	IFN-1a (*n* = 21)	GA (*n* = 24)	NTZ (*n* = 12)
Age, mean ± SEM	39.25 ± 0.5	35.05 ± 0.33	44.05 ± 0.43	37.05 ± 0.49	38.92 ± 0.75
Sex (men/women)	6/14	8/14	3/18	6/18	4/8

### IL-27 is expressed in the MS brain by astrocytes

**Fig. 4:**
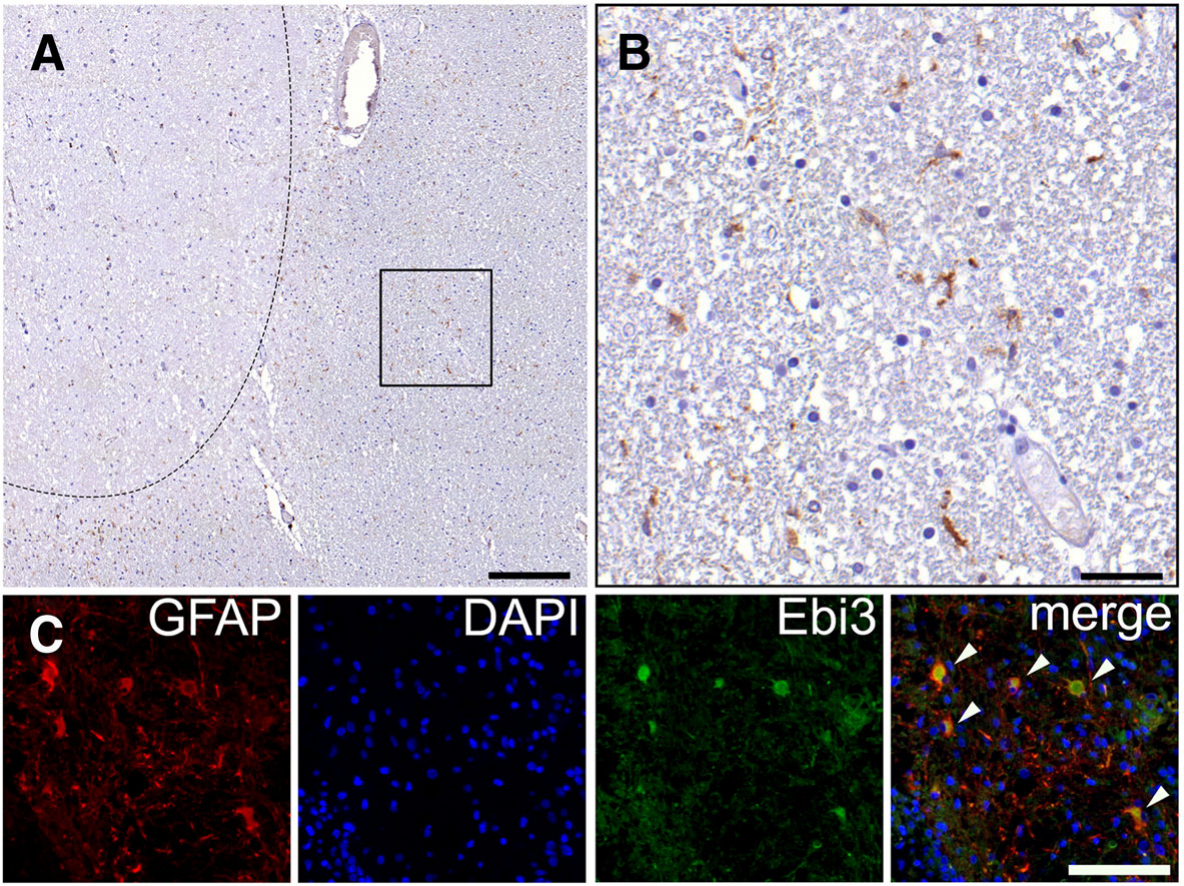
IL-27 expression in MS plaques. **a** A chronic active lesion of a MS patient autopsy was stained for IL-27 using anti-EBI3 antibody visualized with DAB. The *dashed line* indicates the lesion border. **b** Enlarged detail crop from square region in **a**. **c** Representative fluorescence immunohistochemistry image of a biopsy showing an early active MS lesions co-immunostained for EBI3 (*green*) and astrocytes (GFAP, *red*). Nuclei were counterstained using DAPI (*blue*). *Arrowheads* in the merged image indicate EBI3, GFAP double positive cells. Scale bars: **a** = 200 μm; **b** = 40 μm; **c** = 100 μm

We next investigated if IL-27 could be detected in human MS lesions similarly as has been previously described in autopsy MS lesions [12]. First, we assessed IL-27 expression by bright field immunohistochemistry using an antibody directed against EBI3 (a subunit of IL-27) on an autopsy MS lesion showing a hypocellular center and a more hypercellular rim (referred to as chronic-active lesion stage according to [15]). EBI3 immunoreactivity was mostly observed at the rim of the demyelinated lesion (Fig. 4a). At higher magnification, the morphology of EBI3 positive cells resembled astrocytes (Fig. 4b) which was further confirmed by fluorescence co-staining for EBI3 together with astrocyte specific marker GFAP (data not shown).

The notion that EBI3 immunoreactivity was prominent in more active lesion areas together with the observation that IL-27 expression was noted at high levels in the CSF at early stages of the disease prompted us to investigate EBI3 expression in histopathological early active lesion stages in MS biopsies [16, 17]. We focused here on pattern II early active MS lesion in biopsies, as described previously [18], derived from patients with relatively short disease duration with a mean duration of 5 years (±2.5 years) (Table 3). In these biopsies, we could detect EBI3 signal (although at variable intensity) that mostly co-localized with GFAP-expressing astrocytes in five out of six analyzed lesions (Table 3), one example being shown in Fig. 4c. Altogether these analyses suggest that astrocytes may represent the main cellular source of IL-27 in MS lesions which is in line with previous observations [12].

**Table 3:** Description of cerebral biopsies

Samples	Gender	Age [years]	DD [years]	Lesion type	Pattern
MS-1	W	52.69	12.74	EA	II
MS-2	W	NA	NA	EA	II
MS-3	W	73.17	0.43	EA	II
MS-4	M	30.89	0.06	EA	II
MS-5	W	35.66	2.89	EA	II
MS-6	W	54.69	8.99	EA	II

*W* women, *M* men, *NA* not available, *EA* early active lesion

## Discussion

This study shows that patients suffering from RRMS exhibit increased IL-27 expression solely in the CNS compartment as its levels were increased in RRMS patients compared to controls in CSF but not in sera. Interestingly, IL-27 was positively correlated with inflammatory markers in particular white blood cell count and OB positivity. The observation that IL-27 positively correlated with OB in ON patients points toward a role for IL-27 in early inflammation. Biochemical changes of the CSF compartment can be considered as a marker of the CNS inflammation due to its anatomical contact with the brain interstitial fluid. This corroborates with immunohistochemistry staining on cerebral MS biopsies of early active lesions, where astrocytes are found at the highest level. This is in agreement with CSF analysis and highlights a local secretion of IL-27 within the CNS during early neuroinflammation.

IL-27 has been assigned with both pro- and anti-inflammatory functions [19]. The increased levels of IL-27 in the CSF that we observe in RRMS patient could either indicate that IL-27 is implicated in the causality of the disease with a pro-inflammatory role, or in opposition be regarded as a secondary host response that would be held to fight an ongoing disease. We are in favor of a regulatory role of IL-27, in line with murine models. Our observations that higher IL-27 levels in the CSF are not positively but on the contrary possibly negatively correlated with higher EDSS scores support this hypothesis. IL-27 levels could be regarded as part of a regulatory response held to counterpart the initial inflammation, but that might however be insufficiently maintained with disease evolution. An imbalance between inflammatory and regulatory signals could thus be linked with disease progression. This hypothesis is supported by the ability of IFN-β [6] and glatiramer acetate [8], two MS drug-modifying treatments, to stimulate IL-27 production which could itself be held as a marker to identify responders to those treatments [6]. Moreover, the elevated CSF IL-27 levels can be compared to the increased CSF IL-10 levels, a recognized anti-inflammatory cytokine, during RRMS [20]. IL-10 levels are not only increased in the CSF of MS patients compared to healthy subjects but also higher at an active stage compared to a relieving stage in MS patients [21], comforting the idea that the development of a regulatory response is present at early stages during neuroinflammation.

IL-27 is composed of two subunits, EBI3 and p28. A specific ELISA kit detection for IL-27 has been used that specifically detects the heterodimeric cytokine IL-27, but an antibody specific only for EBI3 was used for histology. While p28 is specific for IL-27, the EBI3 combined p35 composes IL-35 cytokine. We however believe that EBI3 staining is representative of IL-27 cytokine as it is typically produced by antigen-presenting cells (APCs) [22] while IL-35 is produced by lymphocytes, in particular Foxp3^+^ regulatory T-cells [23, 24] and regulatory B-cells [25]. In the CNS, we observed EBI3 expression in astrocytes but not in lymphocytes in MS plaques thus supporting our interpretation that IL-27 but not IL-35 is produced by astrocytes in the context of MS disease. In one of the six biopsies, we did not detect EBI3. This could indicate that EBI3 might not be uniformly expressed within the plaques. Alternatively, the detected epitope of EBI3 might be vulnerable to tissue fixation-related effects resulting in antigen masking that prevents antibody binding on paraffin-embedded tissue as used for this study; overfixation might indeed lower epitope recognition by 2G4H6 mAb. Further studies are thus needed to corroborate our histological findings.

IL-27 has anti-inflammatory properties during autoimmune diseases. We propose that local production of IL-27 by astrocytes highlights the induction of a regulatory response that promotes inflammation’s resolution that is the hallmark of early MS lesions. Lymphocytes, in particular infiltrating CD4^+^ T cells express highest levels of IL-27 receptor in the CNS, in a EAE mouse model [10] and in post-mortem tissue analysis [12] suggesting that IL-27 produced locally at the site of inflammation may regulate T cell responses during neuroinflammation.

## Conclusions

Our study provides additional evidence that IL-27 may contribute to autoimmune processes in MS patients.

## Abbreviations

CNS: Central nervous system
CSF: Cerebrospinal fluid
EAE: Experimental autoimmune encephalomyelitis
MS: Multiple sclerosis
OB: Oligoclonal IgG bands
ON: Isolated optic neuritis
RRMS: Relapsing remitting multiple sclerosis
